# Development and Usability Evaluation of a Desktop Software Application for Pain Assessment in Infants

**DOI:** 10.1080/24740527.2018.1540261

**Published:** 2018-11-14

**Authors:** Amos S. Hundert, Marsha Campbell-Yeo, Harrison R. Brook, Lori M. Wozney, Kelly O’Connor

**Affiliations:** a Centre for Pediatric Pain Research, IWK Health Centre, Halifax, Nova Scotia, Canada; b Novum Scientific, Antigonish, Nova Scotia, Canada; c School of Nursing, Centre for Transformative Nursing and Health Research, Dalhousie University, Halifax, Nova Scotia, Canada; d Department of Pediatrics, Dalhousie University, Halifax, Nova Scotia, Canada; e Department of Psychology and Neuroscience, Dalhousie University, Halifax, Nova Scotia, Canada; f Department of Geosciences, University of Edinburgh, Edinburgh, UK; g Centre for Research in Family Health, IWK Health Centre, Halifax, Nova Scotia,Canada

**Keywords:** infant, pain, pain measurement, Premature Infant Pain Profile, software, usability

## Abstract

**Background:**

Pain assessment is a key component of pain management and research in infants. We developed software to assist in coding of pain in infants called PAiN (Pain Assessment in Neonates).

**Aims:**

The aims of this study were to evaluate the usability of PAiN in terms of effectiveness, efficiency, and satisfaction among novice and expert users and to compare the efficiency and satisfaction of PAiN to existing software for coding of infant pain among expert users.

**Methods:**

A quantitative usability testing approach was conducted with two participant groups, representing novice and expert end-users. Testing included an observed session with each participant completing a pain assessment coding task, followed by administration of the Post Study System Usability Questionnaire and Desirability Toolkit. For comparison, the usability of existing coding software was also evaluated by the expert group.

**Results:**

Twelve novice and six expert users participated. Novice users committed 14 noncritical navigational errors, and experts committed six. For experts, the median time for completing the coding task was 28.6 min in PAiN, compared to 46.5 min using the existing software. The mean Post Study System Usability Questionnaire score among novice (1.89) and expert users (1.40) was not significantly different (*P* = 0.0917). Among experts, the score for the existing software (4.83) was significantly (*P* = 0.0277) higher compared to PAiN (1.40). Lower scores indicate more positive responses.

**Conclusions:**

Users were highly satisfied with PAiN. Experts were more efficient with PAiN compared to the existing software. The study was critical to ensuring that PAiN is error free and easy to use prior to implementation.

## RÉSUMÉ

**Contexte**: L’évaluation de la douleur est une composante clé de la prise en charge et de la recherche sur la douleur chez les nourrissons. Nous avons développé un logiciel pour aider à codifier la douleur chez les nourrissons appelé PaiN (*Pain Assessment in Neonates*).

**But**: Évaluer l’utilisabilité de PaiN en termes d’efficacité, d’efficience et de satisfaction auprès d’utilisateurs novices et experts, et comparer l’efficacité de PaiN et la satisfaction d’utilisateurs experts au logiciel existant pour la codification de la douleur chez les nourrissons.

**Méthodes**: Une étude quantitative pour tester l’utilisabilité a été menée auprès de deux groupes de participants, représentant des utilisateurs finaux novices et experts. Le test comprenait l’observation d’une session où chaque participant devait effectuer une tâche de codification de l’évaluation de la douleur, suivie de l’administration du *Post Study System Usability Questionnaire* et du *Desirability Toolkit*. À des fins de comparaison, l’utilisabilité d’un logiciel de codification existant a aussi été évaluée par le groupe expert.

**Résultats**: Douze utilisateurs novices et six utilisateurs experts ont participé à l’étude. Les utilisateurs novices ont commis 14 erreurs de navigation non critiques, tandis que les experts en ont commis six. Pour les experts, le temps médian pour effectuer la tâche de codification a été de 28,6 minutes pour PaiN, comparativement à 46,5 minutes lorsque le logiciel existant était utilisé. Le score moyen obtenu pour le *Post Study System Usability Questionnaire* chez les utilisateurs novices (1,89) et les utilisateurs experts (1,40) n’était pas significativement différent (p=0,0917). Parmi les experts, le score pour le logiciel existant (4,83) était significativement (p=0,0277) plus élevé comparativement à PaiN (1,40). Les scores plus faibles indiquent davantage de réponses positives.

**Conclusions**: Les utilisateurs étaient très satisfaits de PaiN. Les experts ont été plus efficaces avec PaiN qu’avec le logiciel existant. L’étude était d’importance priomordiale pour s’assurer que PaiN ne contienne aucune erreur et soit facile d’utilisation avant sa mise en oeuvre.

## Introduction

Infants being cared for in hospital receive frequent painful procedures, with preterm infants enduring on average 12 procedures daily.^1–3^ These procedures, such as heel pricks and venipunctures, are painful and are associated with immediate and long-term negative outcomes.^4–15^ Unfortunately, both undertreatment and underestimation of pain in neonatal research and clinical care are common problems, with pain treatments administered for less than half of medical procedures performed.^3,16–19^ Though effective treatments do exist for the reduction and alleviation of infant pain, knowledge gaps regarding the most effective and safe methods of pain relief remain. As a result, there is a need for continued research.^9,20^

Pain assessment is a key component of pain management and research. However, reliable pain assessment is challenging in infants because they cannot verbalize their pain.^9,21^ Numerous infant pain assessment tools have been developed, but no recommended gold standard tool has emerged.^21–23^ Methods used to assess pain generally include physiologic or behavioral indicators or a combination of both. Indicators include specific facial movements associated with pain expression, activity level, and vital signs.^22^

One of the most frequently used measures is the Premature Infant Pain Profile (PIPP).^24^ The PIPP has been the most rigorously evaluated neonatal pain assessment tool in psychometric studies and demonstrates good reliability and validity.^25^ The PIPP is a multidimensional measure composed of three facial movements (brow bulge, eye squeeze, nasolabial furrow), two physiological indicators (heart rate and oxygen saturation), and two contextual indicators (gestational age and behavioral state). Each of the seven indicators is scored on a four-point scale (0–3) that reflects increasing change from baseline values. Scores are summed for a total score (maximum 21), representing pain intensity. Recently the PIPP–Revised has been developed, which includes minor scoring and instructional revisions based on feedback to improve ease of clinical use and validity.^26^

The PIPP is used in both clinical practice and research.^27^ When used for research purposes, PIPP scores are often calculated retroactively, using collected physiological data and up-close digital video recording of the infant’s face. Physiological indicators are calculated based on a change from baseline; thus, collection of typical resting baseline measures is essential. Baseline phases also include an assessment of the infant’s behavioral state (e.g., active and awake) during the first 15 s of the baseline. Corrected gestational age on the day of the procedure is taken from the chart and recorded. Though baseline facial actions are not required to calculate PIPP scores, they can be helpful to determine any group difference preceding the procedure and are generally collected. PIPP assessments for research are typically conducted at multiple phases around a painful procedure. For example, pain intensity can be measured using a PIPP score immediately following the procedure (e.g., needle stick) and throughout the procedure (e.g., squeezing of the foot for blood collection), as well as during regulation or time to recover following the procedure. Each phase is generally composed of epochs to help standardize response across studies, and epochs are typically 30-s time intervals. The amount of time that each of the three PIPP facial pain indicators is present is recorded during the epoch.^24,26^ To calculate accurate facial response, the epoch is viewed three times, once to code each facial indicator of pain individually (i.e., brow bulge, eye squeeze, nasolabial fold). To facilitate assessment and data management of PIPP scores, computer software is often used to assist in coding the facial indicators of pain from the video recordings. Yet there is no standard software system for coding assessment and scoring of the PIPP. Modern software capabilities present new opportunities to improve on outdated software to increase coding efficiency, improve data quality, and create a more user-friendly experience.

We aimed to develop a modern software application for facial coding and data management. In order to ensure that the software would meet the needs of prospective users, we conducted a formal usability evaluation. Usability evaluation is a process that assesses the capacity of a system to effectively, efficiently, and enjoyably carry users through tasks and is a critical component of user-centered development.^28–31^ Usability testing assesses the ability of the software to fit users’ needs, meet industry standards for design and functionality, and be usable in the environment in which it will be deployed.

### Objectives

The purpose of this study was to develop and evaluate a new software program called PAiN (Pain Assessment in Neonates). The objectives were to (1) evaluate the usability of PAiN in terms of effectiveness, efficiency, and satisfaction among novice and expert users and (2) compare the efficiency and satisfaction of PAiN to existing software for facial coding of infant pain among expert users.

## Methods

### Study design

A quantitative usability evaluation was conducted with two participant groups, representing novice and experienced end-users. Testing included observed usability evaluation sessions and post study questionnaires. Three usability attributes were evaluated: (1) effectiveness, (2) efficiency, and (3) satisfaction.^31–33^ For comparison, the usability of the existing coding software currently in use at the IWK Health Centre was also assessed. The study protocol and procedures were reviewed and approved by the IWK Health Centre Research Ethics Board.

### Software

#### PAiN software

PAiN includes two separate programs, the PAiN coding program and the PAiN administrator program. The software functions on both Apple OS X and Microsoft Windows operating systems. The usability evaluation was conducted on the coding program only, with both the OS X and Windows versions.

The PAiN coding program uses prerecorded video of an infant’s face and guides users through coding indicators of pain. When the pain indicator is present in an infant while coding, the user holds the spacebar key for the duration of time that the pain indicator is observed. The coding key is accurate to 200 ms. All buttons in PAiN include an associated keyboard shortcut. The software currently includes the PIPP and PIPP-R measures. PAiN allows users to create custom coding templates for specific research study parameters. Templates specify the pain measure used, as well as the number of coding intervals and their length of time for each painful procedure.

**Figure 1.**
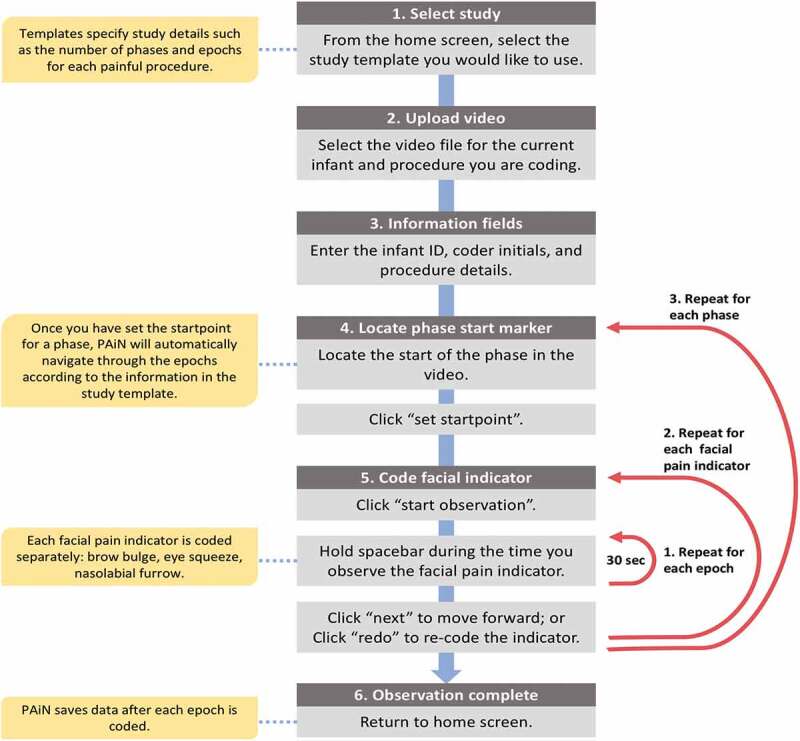
The infant pain indicator coding process in the PAiN software.

**Figure 2.**
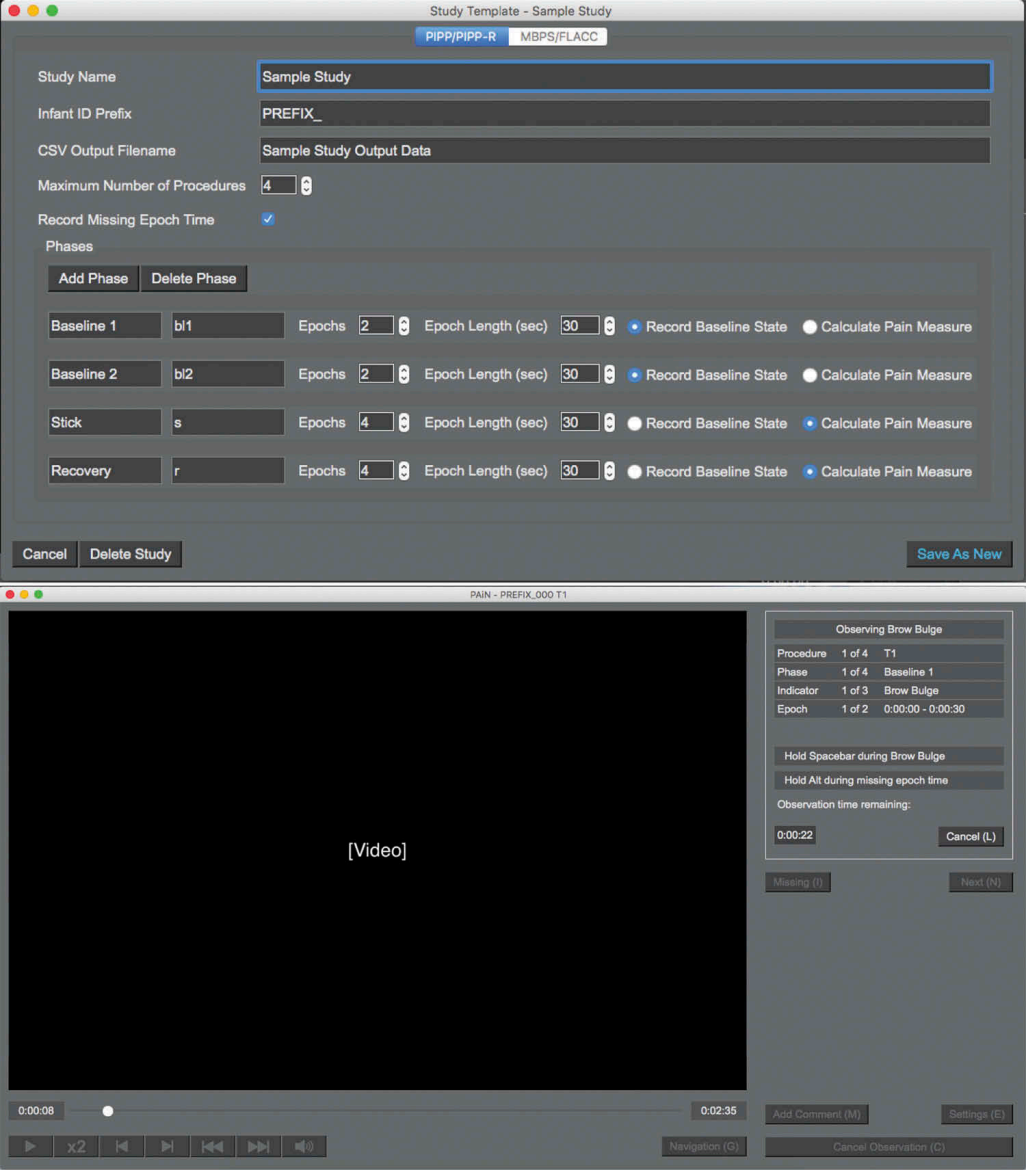
The PAiN software study template window and coding window, following the usability evaluation.

During coding, the start of each phase (e.g., baseline phase) is marked in the infant video by a colored marker flashed on the screen. In PAiN, the user navigates using the built-in video player to the colored marker corresponding to the current phase, pauses the video, and specifies the start point of the phase. PAiN then automatically navigates the user through the video for the phase, based on the study template information, as the user codes each epoch and pain indicator. The user then navigates to the colored marker corresponding to the next phase and repeats the process until coding is complete. Coding output data are saved as CSV files. See Figure 1 for a flow diagram demonstrating the coding process in the PAiN software and Figure 2 for screenshots of the software.

The PAiN administrator program includes data management and analysis features and is designed to be used by those providing study oversight. The software incorporates collected physiological (oxygen saturation, heart rate) and contextual (age) data with the coded behavioral pain indicator data to calculate overall PIPP or PIPP-R scores. Users upload the physiological data as CSV files including time stamps of the PIPP/-R phases and subject IDs. In combination with the facial coding data captured in PAiN, the administrator program calculates overall PIPP/-R scores. Usability testing was not conducted on the administrator software because it is used by a limited number of staff to compile data, with minimal user interface features.

The PAiN software is written in the Python programming language and uses the open source library PySide, a Python interface for Qt. Qt is a cross-platform tool for building graphical user interfaces that appear as native applications on Mac, Windows, and Linux operating systems. PAiN also uses the free and open source VLC Media Player as its audio and video back-end. To prevent loss of data, the data output files are saved each time a data point is recorded by the user.

#### Existing software

The existing software being used at the IWK Health Centre was used for comparison. The software is a proprietary system developed during the 1990s in the BASIC programming language (hereinafter referred to as the BASIC software). The BASIC coding software uses a command-line interface (text based), which requires users to input text commands and necessitates training for users to become familiar with the interface. The BASIC software is not compatible with modern (64-bit) computer operating systems, requires frequent input from users, does not support video playback within the software, does not capture precise time intervals while coding, and requires that data outputs be transcribed. Time intervals are manually started and stopped by the user; and as a result, they are not necessarily an exact 30 s and not necessarily the same precise time interval when coding each of the three PIPP pain indicators separately. Coders use video playback software for the infant video file, in combination with the coding software, which displays the length of time a key is pressed over a time interval, and Excel for transcription of the results.

### Participants

Two convenience samples of IWK Health Centre staff were recruited for the study: Health Centre staff (novice users) and staff with infant pain assessment training (expert users). The IWK Health Centre is a tertiary-level hospital specializing in women and children’s health in Nova Scotia, Canada. The two groups represented typical end-users of the software and allowed for evaluation of the new software compared to the BASIC software.

Previous research indicates that the majority of usability problems in software for desktop operating systems are identified by the first five to ten participants; thus, we aimed to recruit ten participants in each sample (expert and novice users).^29,34,35^

All participants were required to be (1) a current or past employee, volunteer, trainee, or affiliated scientist of the IWK Health Centre for a minimum of 6 months; (2) enrolled in or have completed a postsecondary degree; and (3) familiar with general research and coding methods. In addition, those in the expert sample were required to have experience in neonatal pain research, including training and use of the BASIC coding software. All expert users received prior manualized training and had experience coding for existing research studies, which included regular assessment of intra- and interrater reliability of their coding data.

### Procedure

#### Usability session

All usability sessions were facilitated by one member of the research team (ASH). Participants were informed that their infant coding data would not be analyzed or evaluated in any way and would be deleted following the session. Upon completion of the informed consent process, participants answered a brief demographic and computer use questionnaire before beginning the session. All participants conducted the coding using the same facial infant video file input, and the computer screen was video recorded during the session. The session procedures while using the software varied for the study groups. Procedures unique to each study group are detailed in the following sections. For all participants, the facilitator recorded any navigational or software errors as well as general observations as they used the software. Participants completed the Post Study System Usability Questionnaire (PSSUQ) and the Desirability Toolkit with the facilitator outside of the room.

#### Novice group

In alternating order, participants were assigned to test the PAiN software on a computer running either the Microsoft Windows 7 or Apple OS X 10.11 operating system. First, each participant received a 10-min tutorial from the facilitator. The tutorial included an overview of infant pain assessment and the PIPP measure and a demonstration of PAiN features and functionality. Following the tutorial, the participant was instructed to open PAiN and begin by selecting the usability testing coding template and infant video file. All novice participants used a study template, which included three phases: baseline, stick, and recovery phases. Each phase included two 30-s epochs. Given that novice users did not have previous training in identifying infant pain indicators, they did not code using the BASIC software. The focus in the novice group was ensuring that PAiN was easy to use and understand among users with no previous experience. Efficiency was compared between the two systems in the expert group because the expert users were trained and reliable coders. This provided real-world estimates of coding time among end-users.

#### Expert group

Expert participants were asked to code an infant video to determine behavioral pain response during a medically indicated painful procedure using both the BASIC software and PAiN. The software used first was selected in alternating order. Experts used the Windows operating system for both software systems. Prior to using PAiN, experts received the same demonstration of PAiN features and functionality as the novice users and were then instructed to open the software and select the study template and infant video file. Experts used a study template that matched the prior training they received in the BASIC software. The template consisted of four phases: baseline 1, baseline 2, stick, and recovery. Each baseline included two 30-s epochs. The stick and recovery phases included four 30-s epochs. Overall, experts spent 6 min coding for each pain indicator and 18 min coding in total. Expert users were assigned a template consisting of four phases in order to align the coding with the training they had previously received. For novice users, the shorter coding time consisting of three phases was adequate to evaluate the usability of the PAiN software. Expert users completed the PSSUQ and Desirability Toolkit following use of each software. Errors were not recorded for the BASIC software.

### Measures

#### Demographic and computer use questionnaire

Demographic information collected included age categories, sex, years of postsecondary education categories, and occupation at the IWK Health Centre. Computer use information collected included investigator-developed questions on preferred operating system, the frequency of use of software for data collection and analysis, and two questions on computer proficiency.

#### Errors committed (effectiveness)

Errors committed by each participant were noted by the facilitator during the session and verified by reviewing the recorded screen playback file of all sessions. Errors were categorized as user navigational errors or system errors and as critical or noncritical.^36–38^ Critical errors were considered to be those that resulted in the user being unable to complete the task. Noncritical errors are those that users self-correct from. Users were not necessarily aware of errors committed.

#### Task time (efficiency)

The total time the expert users spent coding with each software system was determined from the screen recordings. The time was calculated from when the coding software was first opened to when all coding activities were completed. Because novice users did not have any prior experience coding and did not use the BASIC software to provide comparison, their coding time was not calculated.

#### PSSUQ (satisfaction)

The PSSUQ is a widely used usability assessment questionnaire with strong psychometric properties.^39–41^ It is a 19-item questionnaire with a seven-point scale. The PSSUQ yields an overall score and can be divided into three subscales: System Usefulness, Information Quality, and Interface Quality. The PSSUQ has excellent reliability, with an alpha coefficient of 0.97. It is sensitive to user group and system differences and valid, based on significant correlation with other measures of user satisfaction. Data support the use of partially completed questionnaires when calculating scale scores, with no significant differences between complete and incomplete questionnaire scale scores.^40^ Overall and scale scores are calculated as the mean response to the included items.

#### Desirability Toolkit (satisfaction)

The Desirability Toolkit is a method of assessing user satisfaction, designed to be a fast and enjoyable alternative to standard questionnaires.^42^ It contains a list of 118 product reaction words that could be used to describe a system (e.g., *organized, stressful*). The words can be modified to best describe the system being evaluated and should maintain a ratio of approximately 60% positive and 40% negative words. See Table 1 for the list of words chosen for this study. Each participant received the words in a different random order. Participants were asked to select the five words that best describe the system they had used or how using the system made them feel.

### Analysis

Data for each participant group, as well as for the BASIC software and PAiN, were analyzed separately. Demographic, task time, errors committed, and desirability toolkit results were analyzed using descriptive statistics to explore distribution and frequency. PSSUQ overall and scale scores were calculated as the mean response to the included scale items. Partially completed questionnaires were included in calculating the scale scores. Nonparametric tests were used to determine whether the mean PSSUQ overall score differed between expert and novice groups using PAiN (Mann-Whitney U test), as well as between expert scores for PAiN and the BASIC software (Wilcoxon signed-rank test). A two-sided *P* value of <0.05 was considered statistically significant and analyses were conducted using STATA 13.^43^

### Minimizing potential conflicts of interest

The study design, data management and analyses, and interpretation of the results were overseen by a co-investigator (LMW) with no conflict. The data analysis plan was established a priori. The usability sessions followed a predetermined format and included primarily quantitative measures as opposed to qualitative interviews in order to minimize the potential for bias introduced by the facilitator during the sessions. Questionnaires were completed without the facilitator present and placed in an opaque envelope before being delivered to a member of the research team (KO) with no conflict, who transcribed the paper questionnaire results. The data were not accessible to any other member of the study team until after all participants completed the usability session.

## Results

### Participant characteristics

Usability sessions were completed during May and June 2016. Overall, 12 novice and six expert users participated. All experts and majority of the novice users were female, with most participants between 21 and 30 years of age. Novice users had a greater number of postsecondary education years compared to expert users. Two thirds of expert and three quarters of novice users preferred using the Apple OS X operating system compared to Microsoft Windows. See Table 2 for participant group characteristics and computer preference and competency.

**Table 1.** Demographic characteristics and computer preferences among expert and novice study groups.

	Study group	
Characteristic	Expert users (*n* = 6), *n* (%)	Novice users (*n* = 12), *n* (%)
Demographic characteristics		
Sex		
Male	0	2 (17)
Female	6 (100)	10 (83)
Age (years)		
≤20	1 (17)	0
21–30	5 (83)	8 (67)
31–40	0	2 (17)
≥41	0	2 (17)
Postsecondary education (years)		
≤4	3 (50)	2 (17)
5–6	2 (33)	2 (17)
7–10	1 (17)	7 (58)
≥11	0	1 (8)
Occupation at IWK Health Centre		
Research assistant	2 (33)	1 (8)
Trainee	4 (67)	0
Graduate trainee	0	4 (33)
Nurse	0	2 (17)
Research coordinator	0	4 (33)
Scientist	0	1 (8)
Computer preferences and proficiencies		
Preferred computer operating system		
Microsoft Windows	2 (33)	3 (25)
Apple OS X	4 (67)	9 (75)
Can usually deal with difficulties encountered when using a computer		
Agree	1 (17)	2 (17)
Somewhat agree	3 (50)	9 (75)
Neutral	0	1 (8)
Somewhat disagree	1 (17)	0
Disagree	1 (17)	0
Find working with computers very easy		
Agree	0	4 (33)
Somewhat agree	5 (83)	6 (50)
Neutral	0	1 (8)
Somewhat disagree	1 (17)	1 (8)
Disagree	0	0
Use software for data collection or analysis		
Daily	1 (17)	0
Weekly	3 (50)	9 (75)
Monthly	2 (33)	2 (17)
Never	0	1 (8)

### Effectiveness

#### User navigational errors

All navigational errors were noncritical. Eight novice users committed 14 navigational errors (mean of 1.2 errors per novice user), and three expert users committed six errors (mean of one error per expert user). Four (33%) novice users and three (50%) expert users committed no navigational errors while using the software. All six errors in the expert group and 11 of the 14 errors in the novice group involved setting the phase start point in the video. Users frequently clicked the “set start point” button prior to navigating to the start point. The start point button was modified following usability testing.

**Figure 3.**
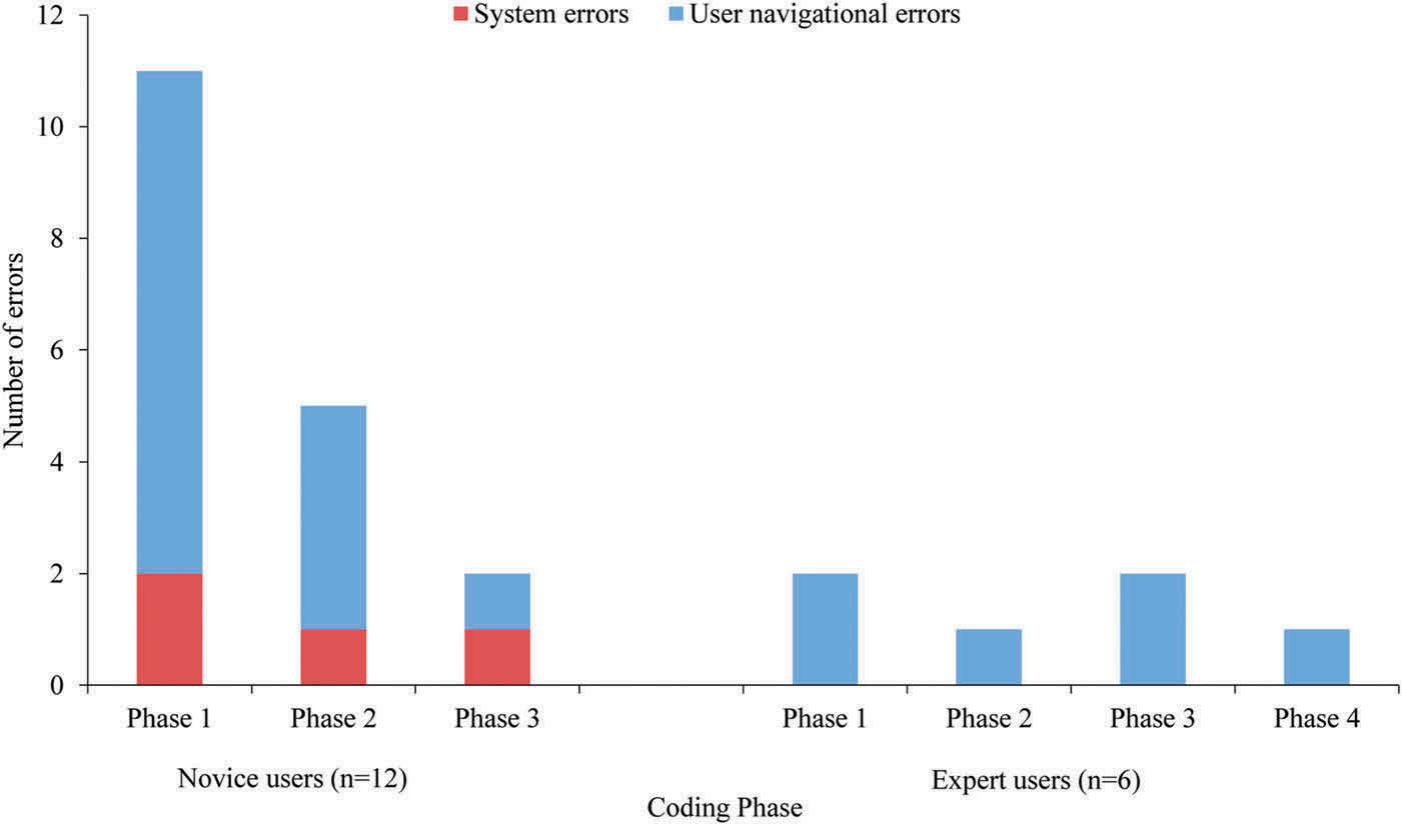
Navigational and system errors committed during each coding phase among novice and expert participants.

Among novice users, the number of errors committed dropped from nine in phase 1 to one in phase 3 (Figure 3). Among expert users, four of the six errors were committed by one user, who committed the same error once in each phase while using the software.

#### System errors

Four system errors occurred during the study among four different participants (mean of 0.3 errors per novice user). All four occurred with novice participants using the OS X PAiN software. The errors occurred while coding a pain indicator and the spacebar was pressed. All four were critical, with PAiN crashing and requiring the user to open and begin again. As a result of these errors, the OS X PAiN software was not used in the expert group. The OS X version continued to be tested in the novice group. Two participants did not continue following the crash, which occurred while coding the last phase (recovery). The two other errors occurred during the first phase (baseline) and the participants restarted the coding task, with no system errors reoccurring. All four users completed the poststudy questionnaires.

Following the usability study, changes were made to prevent the critical error from occurring. All four system errors were determined to be the result of PAiN consuming high levels of memory when several functions were executed at once on an OS X device with low available memory. The source code for PAiN was revised to reduce memory usage, in particular the blocks of code that determine the state of the coding key (spacebar).

### Efficiency

#### Coding time

The template used by expert participants in both software included 18 min of total coding time. The median time from opening the software until completion of coding was 17.9 min less using PAiN compared to the BASIC software. Coding times were less dispersed using PAiN (range: 25.4–30.1 min) compared to the BASIC software (range: 35.1–109.2 min). See Table 3 for coding time descriptive statistics.

**Table 2.** Time spent to complete coding behavioral pain indicators in PAiN and existing software, expert users.

	Time (min) to complete coding task			
Software	Mean	SD	Median	Range
PAiN (*n* = 6)	28.1	1.9	28.6	25.4–30.1
Existing (*n* = 6)	55.4	26.8	46.5	35.1–109.2

PAiN = Pain Assessment in Neonates.

### Satisfaction

#### Post Study System Usability Questionnaire

Nine (38%) of the 24 PSSUQ questionnaire responses (each expert user completed two) included no missing items, and ten (42%) included one missing item. The most common missing item was “the system gave error messages that clearly told me how to fix problems,” missing in 14 (58%) of 24 questionnaires. This is consistent with existing data on PSSUQ response patterns.^40^ Not all participants encountered error messages while using the software, which may explain why many did not respond to the item. Among expert user responses to the PAiN software, three included one missing item and two included two missing items. For expert responses to the BASIC software, three included one missing item and one had two missing items. For novice user responses, five included one missing item, two included two missing items, and one included thre missing items.

Both novice and expert users were highly satisfied with PAiN based on the overall PSSUQ scores. Participants found PAiN to be a useful system with an easy-to-use interface and user information. The mean overall scores for novice and expert users were not significantly different (*P* = 0.0917). In the expert group, the mean overall PSSUQ scores for the BASIC software were significantly (*P* = 0.0277) higher compared to the PAiN software scores. See Table 4 for overall and scale PSSUQ PAiN scores among novice and expert users, as well as expert scores for the BASIC software. PSSUQ score values range from one to seven, with lower scores representing a more positive response. With the BASIC software, the Interface Quality score was notably lower (6.28) than the overall score (4.83). This may have been the result of the line command interface, separate video playback software, and data transcription to Excel. These factors also required the user to interact with additional windows and interfaces while using the software.

**Table 3.** Mean overall and scale scores for the PSSUQ among expert and novice study groups.

	Mean PSSUQ overall and scale scores			
Study group and software evaluated	Overall	System Usefulness	Information Quality	Interface Quality
PAiN software				
Novice users (*n* = 12)	1.89	1.68	2.17	2.01
Expert users (*n* = 6)	1.40	1.38	1.41	1.44
Existing software				
Expert users (*n* = 6)	4.83	4.45	4.52	6.28

PSSUQ = Post Study System Usability Questionnaire; PAiN = Pain Assessment in Neonates.

#### Desirability Toolkit

In the expert user group, participants selected 17 unique words out of 30 total words for PAiN and a further 17 unique words for the BASIC software. All 17 words selected for PAiN had a positive meaning, whereas 16 selected for the BASIC software were negative and one was positive (*familiar*). The most common word selected to describe PAiN was *easy to use*, selected by all six (100%) participants, followed by *straightforward* (four; 67%) and *time-saving* (three; 50%). For the BASIC software, all six (100%) participants selected *dated*, followed by *time-consuming* (five; 83%). The words *annoying, error prone, frustrating*, and *old* were each selected by two (33%) participants.

In the novice user group, a total of 61 words were selected (one participant selected six words), including 28 unique words. They included 25 words with positive meaning and three negative (*stressful, unattractive*, and *sterile*) words. The most frequently selected words were *straightforward* (six; 50%), *easy to use* (six; 50%), *organized* (five, 42%), *useful* (five, 42%), and *efficient* (four, 33%).

Participants in general indicated a positive experience using PAiN based on the selected words. PAiN was described in very systematic or practical terms, with few words selected implying a positive appearance or feeling. Both participant groups selected similar terms to describe PAiN, with *straightforward* and *easy to use* among the most frequently selected words. The expert users selected negative terms to describe the BASIC software, many of which were the antonym of the words selected to describe PAiN (e.g., *time-saving* vs. *time-consuming*).

## Discussion

### Study implications

The usability testing was critical to ensure that the software is a valid method of data collection, error free, and easy to use. Results from this study were used to inform changes to PAiN that increase coding efficiency and usability prior to implementing the software as a research tool. Overall, based on the low number of user errors, low PSSUQ scores, and time experts took to complete coding tasks, the system is acceptable for use in coding infant pain indicators. The changes to PAiN following testing resulted in minor modifications to the software and a second cycle of usability evaluation was not required.

The expert and novice participant groups contributed distinct feedback. Importantly, the novice users did not have expectations for PAiN based around their experience with the existing system.^44^ Novice users were also important in testing whether users with minimum knowledge of infant pain assessment were able to learn to use PAiN with minimal training. Expert participant feedback was critical because they provided insight into the acceptability and validity of the coding process and allowed for the comparison with the BASIC software.

Valid pain assessment is critical to using pain intensity as an outcome in research on pain treatments and mechanisms in infants.^45^ Recently, the American Academy of Pediatrics recommended that more research be conducted on pain reduction strategies and assessment tools in infants.^9^ PAiN software will help facilitate this research. The software will increase the speed of pain assessment data collection, compared to the BASIC software, at the IWK Health Centre and reduce the time required to train coders. By providing an easier to use system for coders, PAiN will also improve data quality, because poor usability is associated with an increase in user errors in medical software.^46–48^ New assessment solutions should be designed to offer a more standardized approach to pain assessment, and PAiN is progressing toward an increasingly automated method.^23,49,50^ The software removes a degree of user control, allowing the user to maintain focus on coding. By automating the process of navigating through the video during coding, PAiN improves data quality by maintaining precise time intervals during the coding of each facial pain indicator. The need for data transcription and the potential for errors associated with transcription are also eliminated.^51,52^ The increased automation removes the potential for individual approaches to coding and variation in protocol between coders. However, this also results in a rigid coding process. There exist over 48 different measures developed to assess infant pain, which include varying approaches to pain assessment.^53^ This research evaluated the usability of software using one well-established approach.^24,26^

### Limitations

The study had limitations. Sample size in the expert group was limited by the number of individuals available with training in the BASIC software and the desired sample size was not reached. We do not believe that this impacted the ability to detect important usability issues, because study participants gave similar feedback and committed similar errors. Additionally, more novice participants were recruited than specified. Other limitations include that no usability session conversation was transcribed and no formal content analysis was conducted. No testing was conducted on other pain measures, though the coding method is similar across pain measures in the software. The BASIC software used as a comparison to PAiN is not a standard tool and may not be generalizable to pain assessment methods used by other research groups.

### Future directions

PAiN will now be implemented as a research tool at the IWK Health Centre. Ongoing evaluation of PAiN during implementation will occur, including analysis of the inter- and intracoder reliability of facial pain indicator scores coded in PAiN. The software is highly adaptable to the addition of different pain measures. Two tools, the Face, Legs, Activity, Cry, Consolability Scale and Modified Behavior Pain Scale, have now been included in the software with the potential for others to be added as needed.^54,55^

Progress on the development of valid fully automated solutions for infant pain assessment is limited by a lack of relevant infant pain data for analysis.^56,57^ We plan to include a data output in PAiN that logs the time intervals (accurate to 200 ms) that each pain indicator is present in the infant video based on the coder input. In the future, these data may be used to inform development of an automated method of infant pain assessment using machine learning techniques.^56^

## Supplementary Material

Supplemental Material

## Appendix

**Table:** Words included in the desirability toolkit for study participants.

Desirability Toolkit word list				
Accessible	Controllable	Flexible	New	Sophisticated
Advanced	Convenient	Fragile	Nonstandard	Stable
Ambiguous	Counterintuitive	Fresh	Not secure	Sterile
Annoying	Creative	Friendly	Not valuable	Stimulating
Appealing	Credible	Frustrating	Obscure	Straightforward
Approachable	Cutting-edge	Fun	Old	Stressful
Attractive	Dated	Hard to use	Ordinary	System-oriented
Awkward	Desirable	High-quality	Organized	Time-consuming
Boring	Difficult	Illogical	Overwhelming	Time-saving
Bright	Distracting	Impressive	Patronising	Tiring
Business-like	Dull	Inadequate	Poor quality	Too technical
Busy	Easy to use	Incomprehensible	Powerful	Trustworthy
Calm	Effective	Inconsistent	Predictable	Ugly
Clean	Efficient	Ineffective	Professional	Unattractive
Clear	Effortless	Innovative	Relevant	Unconventional
Cluttered	Energetic	Insecure	Reliable	Understandable
Comfortable	Engaging	Intimidating	Responsive	Unpredictable
Compatible	Entertaining	Intuitive	Rigid	Unrefined
Compelling	Error prone	Inviting	Satisfying	Usable
Complex	Exciting	Irrelevant	Scientific	Useful
Comprehensive	Expected	Low maintenance	Secure	Vague
Confusing	Familiar	Meaningful	Simple	Valuable
Consistent	Fast	Misleading	Simplistic	
Contradictory	Faulty	Motivating	Slow	
