# Supplementary figures and images for: Development and Usability Evaluation of a Desktop Software Application for Pain Assessment in Infants
Source: Can J Pain. 2018 Nov 14;2(1):302–14. doi: 10.1080/24740527.2018.1540261 (PMC8730649; doi:10.1080/24740527.2018.1540261)

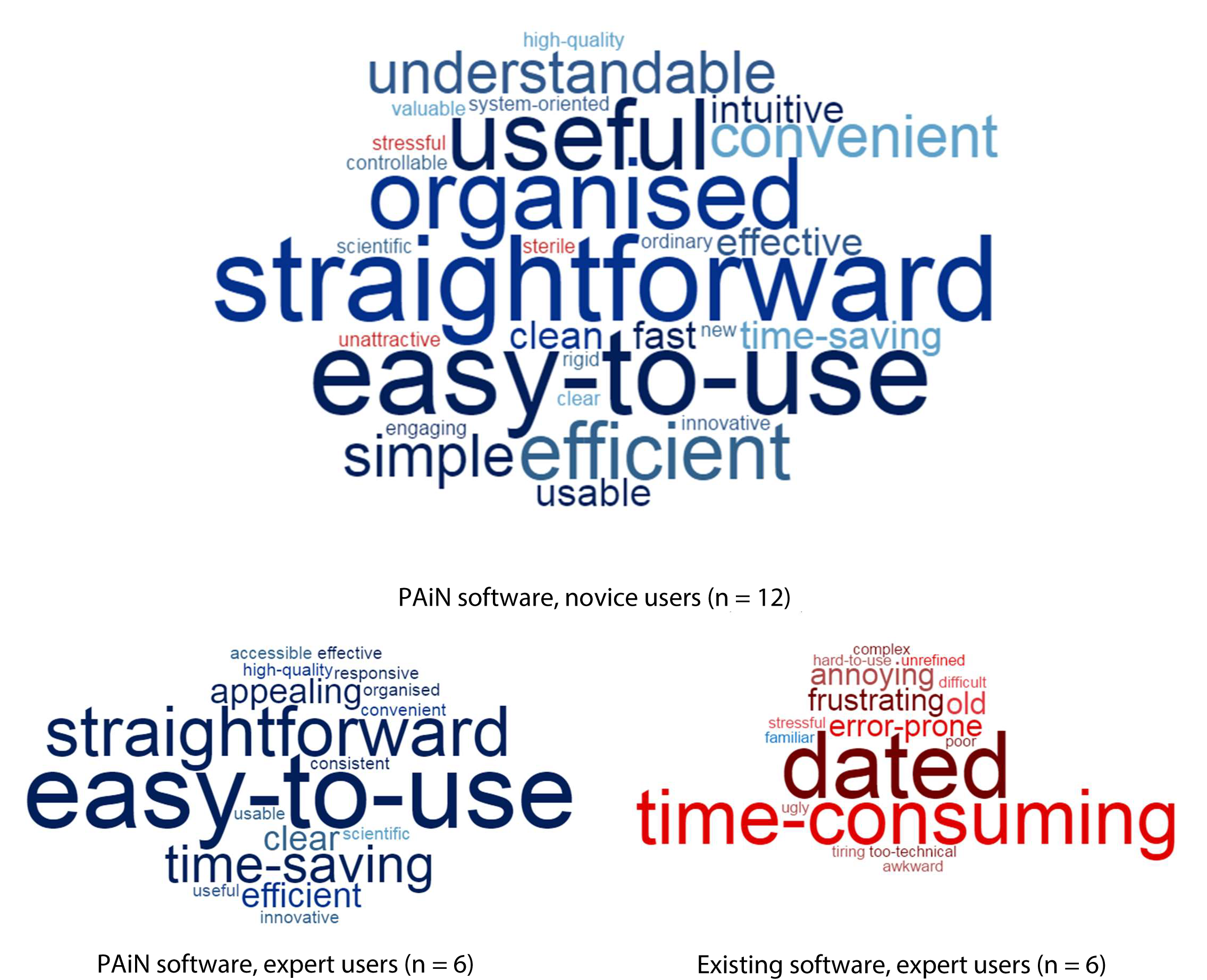

Supplement: Supplemental Material [file UCJP_A_1540261_SM5436.tif]
